# Synthesis and Characterization of Two Sparfloxacin Crystalline Salts: Enhancing Solubility and In Vitro Antibacterial Activity of Sparfloxacin

**DOI:** 10.3390/pharmaceutics16121519

**Published:** 2024-11-26

**Authors:** Wei Sun, Ruili Huo, Jingzhong Duan, Jixiang Xiao, Yan Wang, Xiaoping Zhou

**Affiliations:** School of Pharmaceutical Sciences, Jilin University, Changchun 130021, China; wsun@jlu.edu.cn (W.S.); huoruili0925@163.com (R.H.); duanjz22@mails.jlu.edu.cn (J.D.); xiaojx23@mails.jlu.edu.cn (J.X.)

**Keywords:** sparfloxacin, crystalline salts, pimelic acid, azelaic acid, solubility, antimicrobial activity

## Abstract

**Background**: To improve the solubility and permeability of Sparfloxacin (SPX) and enhance its antimicrobial activity in vitro, two unreported pharmaceutical crystalline salts were synthesized and characterized in this paper. One is a hydrated crystal of Sparfloxacin with Pimelic acid (PIA), another is a hydrated crystal of Sparfloxacin with Azelaic acid (AZA), namely, SPX-PIA-H_2_O (2C_19_H_23_F_2_N_4_O_3_·C_7_H_10_O_4_·2H_2_O) and SPX-AZA-H_2_O (4C_19_H_23_F_2_N_4_O_3_·2C_9_H_14_O_4_·5H_2_O). **Methods**: The structure and purity of two crystalline salts were analyzed using solid-state characterization methods such as single-crystal X-ray diffraction, powder X-ray diffraction, differential scanning calorimetry, thermogravimetric analysis, and infrared spectroscopy. Additionally, the interaction characteristics between two crystal salt molecules were examined by constructing Hirshfeld surfaces and mapping specific real-space functions through Hirshfeld surface analysis. The solubility under physiological conditions, diffusivity across simulated biological membranes, and in vitro antibacterial activity against specific bacterial strains of two crystalline salts were evaluated using established assays, including minimum inhibitory concentration (MIC) tests. **Results**: Single-crystal X-ray diffraction and Hirshfeld surface analysis indicate that SPX forms stable crystal structures with PIA through charge-assisted hydrogen bonds N1-H1e···O10 (1.721 Å, 173.24°), N5-H5a···O11 (1.861 Å, 169.38°), and with AZA through charge-assisted hydrogen bonds N5-H5B···O8 (1.810 Å, 154.55°), N4-H4B···O6 (1.806 Å, 174.97°). The binding sites of two crystalline salts were at the nitrogen atoms on the piperazine ring of SPX. Compared with SPX, the equilibrium solubility of the two crystalline salts was improved by 1.17 and 0.33 times, respectively, and the permeability of the two crystalline salts was increased by 26.6% and 121.9%, respectively. In addition, SPX-AZA-H_2_O has much higher antibacterial activity on *Pseudomonas aeruginosa* and *Bacillus subtilis* than SPX. **Conclusions**: This research yielded the successful synthesis of two crystalline salts of Sparfloxacin (SPX), significantly improving its solubility and diffusivity, and bolstering its antibacterial efficacy against targeted bacterial species. These breakthroughs set the stage for innovative advancements in the realm of antimicrobial drug development.

## 1. Introduction

**Figure 1:**
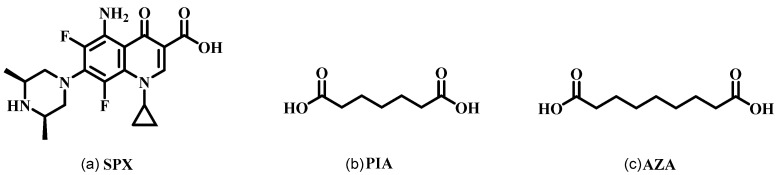
Molecular structure of SPX (**a**), PIA (**b**), and AZA (**c**).

Sparfloxacin (SPX, Figure 1a) is a well-known broad-spectrum fluoroquinolone antimicrobial that inhibits the activity of bacterial DNA gyrase and topoisomerases IV. It exhibits excellent activities against a variety of Gram-positive and Gram-negative bacteria, as well as atypical bacteria in vitro. SPX has shown high efficacy and safety in treating acute sinusitis, chronic bronchitis, and community-acquired pneumonia [1,2,3,4,5,6,7].

However, SPX belongs to Class II drugs in the Biopharmaceutics Classification System (BCS). Despite its high permeability, it has extremely low solubility, which can affect the oral absorption of SPX, thereby impacting its therapeutic efficacy [8,9,10]. This limitation prevents SPX from fully exerting its antimicrobial activity and restricts its usage [6,7]. In the process of drug development, improving the dissolution rate of poorly soluble drugs without compromising their therapeutic potential or other properties is a major challenge [11,12,13]. Due to the development of supramolecular and solid-state chemistry, using crystal engineering to improve the physicochemical properties of active pharmaceutical ingredients (APIs) has become one of the strategies in drug development [14,15,16,17]. Crystal engineering methods, that is, the rational design and synthesis of new crystal forms, meet the needs of the pharmaceutical industry [14,18]. Crystal engineering has been proven to regulate and improve the solubility [19,20,21,22], stability [23,24,25,26], bioavailability [27,28], and other properties of API, thereby enhancing the efficacy and safety of drugs [29,30,31], such as Depakote^®^, Entresto^®^, Suglat^®^, and Steglatro^®^ [32,33]. Crystal engineering techniques can provide a green and efficient method for drug development [34,35].

To improve the solubility of SPX, enhance its antibacterial activity while maintaining its good permeability, we selected two organic dicarboxylic acids, namely Pimelic acid (PIA, Figure 1b) and Azelaic acid (AZA, Figure 1c), to synthesis pharmaceutical crystalline salts of SPX. A series of solid-state characterization techniques were used to confirm the crystal structures, and the intermolecular interactions within the crystal structures were analyzed using Hirshfeld surface analysis. The equilibrium solubility, dissolution rate, permeation amount, and antibacterial activity in vitro of SPX and two new crystals were studied.

## 2. Materials and Methods

### 2.1. Materials

Sparfloxacin (≥99%) and Pimelic acid (≥98%) were purchased from Shanghai Bind Biotech Co., Ltd. (Shanghai, China). Azelaic acid (≥97%) was purchased from Anhui ZeSheng Technology Co., Ltd. (Anqing, China). Organic reagents of HPLC are Fisher chromatographic grade, and other organic reagents are analytical grade. Distilled water was used for all experiments.

### 2.2. Preparation of Crystalline Salts

To prepare SPX-PIA-H_2_O, SPX (0.05 mmol, 19.60 mg) and PIA (0.1 mmol, 16.00 mg) were combined in mixture of 9 mL methanol and 1 mL distilled water, and sufficiently stirred at 50 °C for 3 h. After filtration, the filtrate was evaporated slowly at room temperature. Yellow crystals were obtained through vacuum filtration after 5 days.

To prepare SPX-AZA-H_2_O, SPX (0.05 mmol, 19.60 mg) and AZA (0.05 mmol, 9.40 mg) were combined in mixture of 20 mL methanol and 7 mL distilled water, and sufficiently stirred at 50 °C for 3 h. After filtration, the filtrate was evaporated slowly at room temperature. Yellow crystals were obtained through vacuum filtration after 8 days.

### 2.3. Single Crystal X-Ray Diffraction (SCXRD)

The single crystal X-ray diffraction data of the two crystalline salts were recorded on an X-ray single-crystal diffractometer Bruker APEX-II CCD (Karlsruhe, Germany) with Mo Kα radiation (λ = 0.71073 Å) in multi-scan mode at a temperature of 100 K. The data were collected using the Bruker SMART APEX II diffractometer software (Version 2) and were analyzed and refined using the ShelXL (version 1.0.1678) [36] and Olex 2 (version 1.5) [37] programs. All non-hydrogen atoms were refined with anisotropic displacement parameters. The crystallographic data for the structures were deposited in the Cambridge Crystallographic Data Center with CCDC numbers 2,368,608 for SPX-PIA-H_2_O and 2,368,612 for SPX-AZA-H_2_O.

### 2.4. Powder X-Ray Diffraction (PXRD)

PXRD was performed using an X-ray diffractometer Bruker D8 Advance (Karlsruhe, Germany). The X-ray source was Cu-Kα with a wavelength of 1.540598 Å. The divergence and scattering slits were set to 1°, and the receiving slit was set to 0.15 mm. The voltage and current in the tube were set at 40 kV and 40 mA, respectively. The scanning rate was 5°/min, within the diffraction angle range (2θ) of 5°–50°. The PXRD patterns of crystal structures were simulated using the software Mercury (version 2023.2.0) [38].

### 2.5. Thermal Analysis (DSC and TGA)

DSC was performed using a differential scanning calorimeter model 204 F1 (Selb, Germany). Powder sample was heated in an aluminum pan between room temperature to 300 °C at a rate of 10 °C/min in N_2_ atmosphere.

TGA was performed using a synchronous thermal analyzer NETZSCH STA 2500 (Shanghai, China). The sample was heated from room temperature to 400 °C at a rate of 10 °C/min in N_2_ atmosphere.

### 2.6. High-Pressure Liquid Chromatography (HPLC)

The aqueous solubility of the SPX was determined using Shimadzu LC-20AD high-performance liquid chromatography (HPLC) system. The C18-WR column (WondaSil, 250 × 4.6 mm, 5 μm particle size) was used, and the maximum wavelength (λ_max_) was set to 298 nm. The mobile phase was composed of phosphate solution (6.8 g of potassium dihydrogen phosphate and 3.0 mL of triethylamine, dissolved in water and diluted to 1000 mL) mixed with methanol in a ratio of 45:55. The sample injection volume was 20 µL with a flow rate of 1.0 mL/min. The sample was filtered through a 0.45 µm filter membrane before being analyzed by HPLC, with the run time set for 12 min.

Note: In order to assess the influence of nylon filter membranes on sample integrity, preliminary experiments were carried out using both 0.45 μm nylon filtration and a control without filtration. The outcomes were found to be substantially similar. Consequently, to guarantee the absence of particulates in the samples and to safeguard the instrumentation, 0.45 μm nylon filter membrane has been elected for use in our procedures.

### 2.7. Determination of Equilibrium Solubility

The shake flask method was used to determine the equilibrium solubility of SPX and crystalline salts in pH 1.2 (prepared with hydrochloric acid (36.5%) and diluted), pH 4.0 (prepared with 0.1 M anhydrous citric acid and 0.5 M dibasic sodium phosphate), and pH 6.8 (prepared with 0.2 M potassium dihydrogen phosphate and NaOH) buffer solution and water at 37.0 ± 0.1 °C. The sample was ground and sieved, and then was placed into flasks containing 10 mL of buffer solution. The system was equilibrated for 48 h to obtain a suspension. The suspension was then centrifuged (at 2000 rpm for 10 min), and the supernatant was filtered through a 0.45 μm nylon filter, diluted, and analyzed by HPLC using the chromatographic conditions described above. Measure and compare the pH levels before and after the experiment. Finally, the residue obtained after the equilibrium solubility tests was subjected to PXRD analysis and compared with the pattern obtained before the experiment, respectively. The experiment was carried out in triplicate (*n* = 3).

### 2.8. Determination of Powder Dissolution

Adhering to the paddle method detailed in the Chinese Pharmacopoeia, 2020 edition, dissolution studies were performed on SPX and crystalline salts in various pH buffer solutions (1.2, 4.0, 6.8) and distilled water, utilizing the RC806ADK dissolution apparatus (Tianjin, China). A 30.00-milligram sample of SPX and the equivalent quantity of crystalline salt containing SPX were finely ground and sieved to achieve a homogenous powder. This powder was then transferred into large cups pre-filled with 900 mL of dissolution buffer. The experimental conditions were meticulously controlled, with the temperature maintained at 37 °C and the paddle rotation set at a steady 100 revolutions per minute (rpm/min). At precise intervals—15, 30, 45, 60, 90, 120, 180, 240, 300, and 360 min—1 mL of the solution was carefully extracted, and an equal volume of fresh dissolution buffer was introduced to maintain the system’s integrity. The extracted samples were subjected to filtration through 0.45 μm nylon membrane to ensure clarity, followed by dilution in preparation for high-performance liquid chromatography (HPLC) analysis. Measure and compare the pH levels before and after the experiment. Post-dissolution testing, the residual residue was analyzed using PXRD, and the resulting patterns were compared with those of the crystalline salts prior to the experiment.

### 2.9. Determination of Diffusivity

The diffusivity of SPX and crystalline salts in a pH 7.4 buffer solution (prepared with 0.1 M NaOH and 0.01 M potassium dihydrogen phosphate) was determined using a transdermal diffusion apparatus TPY-2 (Shanghai, China) and a diffusion membrane (MW 14,000, Biosharp, Hefei, China). The pretreatment of the diffusion membrane involved the following steps: (1) boiling for 10 min in a mixture of 2% sodium bicarbonate/1 mM EDTA, followed by rinsing with water; (2) immersion and boiling in water for an additional 10 min; and (3) after removing the water, immersing the diffusion membrane in a solution of 50% ethanol/1 mM EDTA and thoroughly rinsing with water prior to use.

Suspensions of SPX and crystalline salts were prepared in a pH 7.4 buffer solution, respectively. A total of 7 mL aliquot of the suspension was placed into the donor compartment, and 7 mL pH 7.4 buffer solution were placed into the receptor compartment, separated by the diffusion membrane. The temperature was set to 37 °C, and the rotation speed was set to 600 revolutions per minute (r/min). Samples (200 μL) were taken at time intervals of 15, 30, 60, 120, 180, 240, 300, 360, 420, 480, 540, and 600 min, and 200 μL of fresh pH 7.4 buffer solution was replenished. The sample solutions were filtered through a 0.45 μm nylon filter and then diluted for HPLC analysis under the chromatographic conditions previously described. Measure and compare the pH levels before and after the experiment. The experiment was conducted in triplicate (*n* = 3).

### 2.10. Test of Antibacterial Activities

The minimum inhibitory concentrations (MIC) of SPX, PIA, AZA, and crystalline salts against *Escherichia coli*, *Staphylococcus aureus*, *Pseudomonas aeruginosa*, and *Bacillus subtilis* were determined using the microbroth dilution method. The samples were dissolved in DMSO and then diluted in Mueller–Hinton (MH) broth at a ratio of 1:2 in a gradient manner. The bacterial strains were inoculated into tubes containing MH broth and cultured until the bacteria were in the logarithmic growth phase. The bacterial suspensions were added to microplates containing the test samples, with the final concentrations ranging from 0.01 to 1024 μg/mL. Another sterile filter paper was just immersed with DMSO as control. The microplates were placed in a constant temperature incubator at 37 °C and cultured for 24 h. The presence of bacterial growth in each well of the microplates was monitored visually and by spectrophotometry to determine the MIC. The experiment was carried out in triplicate (*n* = 3).

## 3. Results

### 3.1. Single Crystal X-Ray Diffraction (SCXRD)

**Figure 2:**
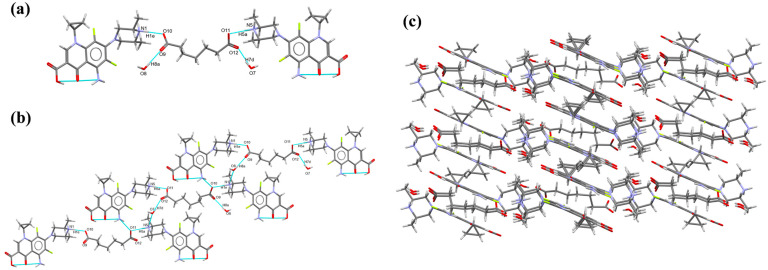
Crystal structure of SPX-PIA-H_2_O: (**a**) asymmetric unit; (**b**) 2D structure; and (**c**) 3D network.

SPX-PIA-H_2_O (2C_19_H_23_F_2_N_4_O_3_·C_7_H_10_O_4_·2H_2_O) crystallizes in the monoclinic crystal system, with the space group P2_1_/c. The asymmetric unit is composed of one SPX molecule, one PIA molecule, and two water molecules, in a stoichiometric ratio of 2:1:2. Two protons from the PIA are transferred to the N1 and N5 atom of the SPX, respectively; within the asymmetric unit, SPX and PIA are interconnected via hydrogen bonds N1-H1e···O10 (1.721 Å, 173.24°) and N5-H5a···O11 (1.861 Å, 169.38°), while water molecules form hydrogen bonds with PIA through O8-H8a···O9 (2.707 Å, 177.30°) and O7-H7d···O12 (1.788 Å, 167.52°)(Figure 2a). Within the crystal lattice, the asymmetric units are linked through a precise network of hydrogen bonds, specifically N3-H3a···O10 (2.026 Å, 153.38°) and N1-H1d···O8 (1.909 Å, 170.48°), which orchestrates the assembly of a two-dimensional structure (Figure 2b). This structured arrangement subsequently integrates to form an intricate three-dimensional network (Figure 2c), showcasing the complexity and beauty of the crystalline architecture. This intricate crystallographic arrangement is meticulously detailed in the Supplementary Tables S1 and S2.

**Figure 3:**
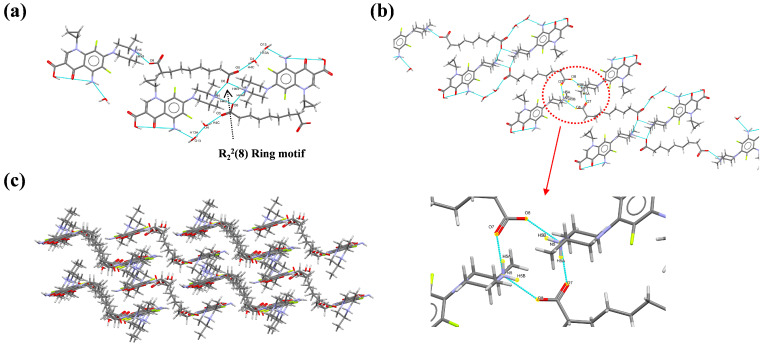
Crystal structure of SPX-AZA-H_2_O: (**a**) asymmetric unit; (**b**) 2D structure; and (**c**) 3D network.

SPX-AZA-H_2_O, with the chemical formula (4C_19_H_23_F_2_N_4_O_3_·2C_9_H_14_O_4_·5H_2_O), crystallizes in the triclinic crystal system, specifically within the P $\bar{1}$ space group. The asymmetric unit comprises four SPX molecules, two AZA molecules, and five water molecules, adhering to a stoichiometric ratio of 4:2:5. Two protons from the AZA are transferred to the N4 and N5 atoms of the SPX, respectively. Within a single asymmetric unit, SPX and AZA are bridged by hydrogen bonds, specifically N5-H5B···O8 (1.810Å, 154.55°) and N4-H4B···O6 (1.806 Å, 174.97°), forming the R_2_^2^(8) ring motif simultaneously. Concurrently, water molecules within the asymmetric unit engage with SPX and AZA through hydrogen bonds, including N2-H2A···O13 (2.143 Å, 167.97°) and O4-H4C···O5 (1.950 Å, 163.80°) (Figure 3a). The asymmetric units are interconnected via hydrogen bonds N5-H5A···O7 (1.799 Å, 169.69°) and N5-H5B···O8 (1.810 Å, 154.55°), forming a 2D network (Figure 3b). This network, through a process of extensive stacking, culminates in the formation of a robust 3D architecture (Figure 3c). This intricate crystallographic arrangement is meticulously detailed in Supplementary Tables S2 and S3.

### 3.2. Powder X-Ray Diffraction (PXRD)

**Figure 4:**
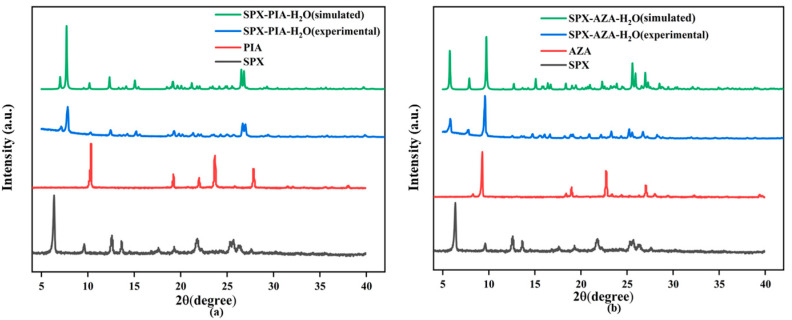
PXRD patterns: (**a**) SPX-PIA-H_2_O; (**b**) SPX-AZA-H_2_O.

The PXRD patterns (Figure 4) indicate that the characteristic diffraction peaks of SPX are located at 6.36°, 9.61°, 12.60°, 13.60°, and 21.80°; the characteristic diffraction peaks of PIA are located at 10.30°, 19.20°, 22.58°, 23.70°, and 27.90°; the characteristic diffraction peaks of AZA are located at 8.29°, 9.29°, 18.40°, 19.67°, 22.70°, and 27.79°. SPX-PIA-H_2_O exhibits new characteristic diffraction peaks at 7.13°, 7.83°, 10.26°, 12.45°, 15.22°, 19.30°, 21.33°, 24.31°, 24.09°, 25.67°, and 26.68°, which are distinct from those of SPX and PIA. SPX-AZA-H_2_O shows new characteristic diffraction peaks at 5.80°, 7.75°, 9.58°, 14.74°, 16.65°, 18.24°, 20.91°, 23.30°, and 26.74°, which are distinct from those of SPX and AZA. Moreover, these are in good agreement with the PXRD patterns simulated by the software Mercury, suggesting that two crystalline salts were successfully synthesized, forming new crystalline phases with high purity.

### 3.3. Thermal Analysis

**Figure 5:**
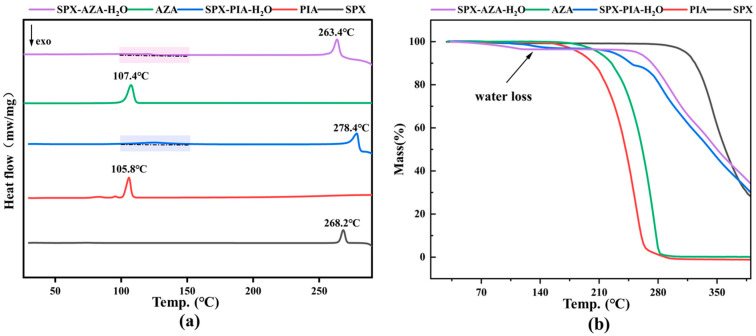
(**a**) The DSC curves; (**b**) the TGA curves.

The endothermic peak (melting point) of SPX-PIA-H_2_O appears at 278.4 °C, which is different to that of both of the individual components SPX (268.2 °C) and PIA (105.8 °C). The melting point of SPX-AZA-H_2_O is 263.6 °C, which is also different to that of both of the individual components SPX (268.2 °C) and AZA (107.4 °C). This indicates that both crystal salts were successfully synthesized and do not contain any single component. Additionally, the DSC curve of SPX-PIA-H_2_O exhibits a single broad endothermic peak between 115 °C and 127 °C (SPX-AZA-H_2_O, 108–120 °C) (Figure 5a), suggesting the loss of water. The weight loss analysis from the TG curves of both is very consistent with the water content, as indicated by the SC-XRD results (Figure 5b). Both results confirm the presence of water molecules in SPX-AZA-H_2_O. Furthermore, the TGA curves of SPX-PIA-H_2_O and SPX-AZA-H_2_O are distinctly different from that of SPX, indicating that after the formation of the crystalline salts, the thermodynamic properties have undergone changes.

### 3.4. Equilibrium Solubility Studies

**Figure 6:**
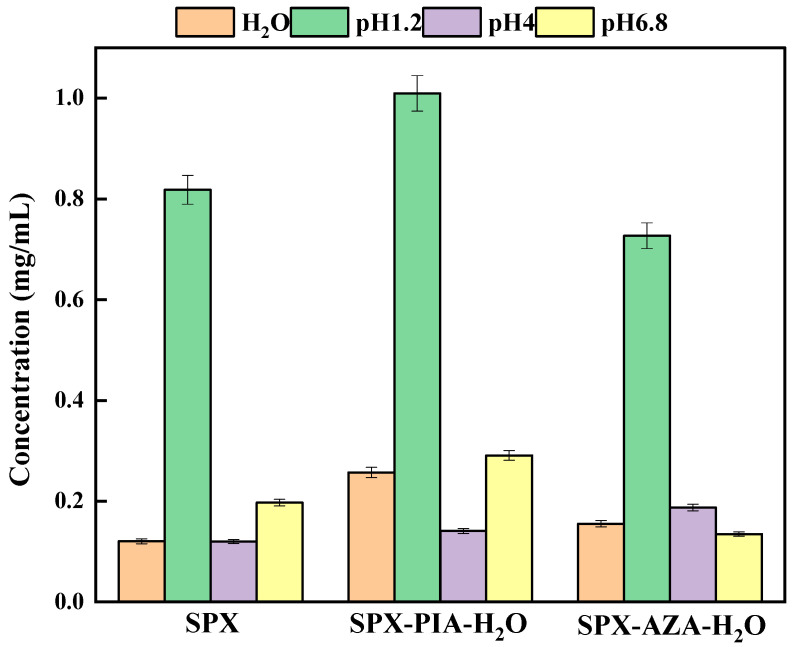
Equilibrium solubility of SPX and crystalline salts.

The equilibrium solubility of SPX in environments with pH values of 1.2, 4.0, 6.8, and in pure water, was measured to be 0.82 mg/mL, 0.12 mg/mL, 0.20 mg/mL, and 0.12 mg/mL, respectively. The synthetic crystalline salt SPX-PIA-H_2_O has been shown to significantly enhance the equilibrium solubility of SPX in these four media, increasing it to 1.23, 1.17, 1.45, and 2.17 times that of SPX, respectively. In contrast, the crystalline salt SPX-AZA-H_2_O only managed to increase the solubility to 1.58 and 1.33 times that of SPX in the pH 4.0 buffer and pure water, respectively (Table S4 and Figure 6). Moreover, its equilibrium solubility was found to be lower in both the pH 1.2 and pH 6.8 environments compared to SPX alone. As shown in Table S5, the pH values of the two crystalline salts in the four solvent media remained essentially constant before and after the experiment. This consistency demonstrates the stability of the crystalline salts in the four solutions throughout the entire experimental process, indicating that the equilibrium solubility results are genuine and reliable. Concurrently, the PXRD patterns of the residual residues from two crystalline salts in four different solvent media were compared with their respective PXRD patterns prior to the experiment (Figure S2). When the solvent medium was pure water, there was a slight change in pH before and after the dissolution test (from 7.22 to 7.86 for SPX-PIA-H_2_O and from 7.22 to 7.58 for SPX-AZA-H_2_O). However, the analysis of the PXRD patterns indicated that despite the pH changes, the crystalline phase of the salts did not alter. This suggests that minor pH variations do not affect the crystalline phase of the salts, and the results of the equilibrium solubility are still reliable. The analysis revealed that the PXRD patterns of the residues were essentially consistent with those obtained before the experiment, with no significant changes in characteristic peaks. This indicates that the dissolution tests did not alter the crystalline phase of the two crystalline salts, which remained relatively stable in the four solvent media and did not affect the determination of equilibrium solubility results. These findings suggest that the equilibrium solubility of SPX can be substantially improved by utilizing PIA as a coformer in the synthesis of the crystalline salt with SPX, highlighting the potential of SPX-PIA-H_2_O as a promising formulation strategy.

### 3.5. Powder Dissolution Studies

**Figure 7:**
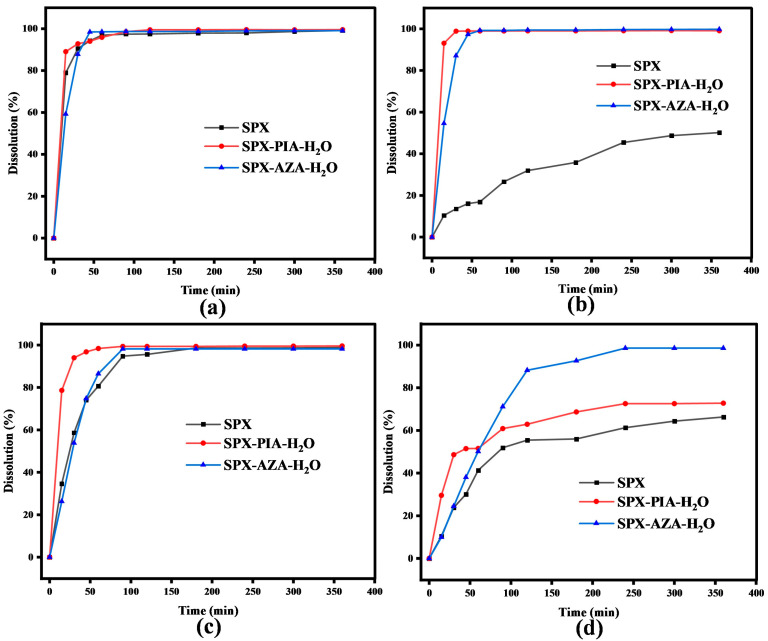
Dissolution curves: (**a**) pH 1.2; (**b**) pH 4.0; (**c**) pH 6.8; and (**d**) pure water.

Under the conditions of 37 °C, the dissolution processes of SPX, SPX-PIA-H_2_O, and SPX-AZA-H_2_O in four different buffer media were assessed (Figure 7). In the pH 1.2 buffer, all compounds achieved over 90% dissolution, with SPX exhibiting a more rapid dissolution rate. In the pH 4.0 buffer, SPX showed poor solubility, dissolving only about 50% within 600 min, while SPX-PIA-H_2_O and SPX-AZA-H_2_O surpassed 95% dissolution, significantly outperforming SPX. In the pH 6.8 buffer, the dissolution rates of SPX-PIA-H_2_O and SPX-AZA-H_2_O were higher than that of SPX, yet no significant difference in dissolution was observed compared to SPX. In pure water, the dissolution rate of SPX was approximately 65%, whereas SPX-PIA-H_2_O and SPX-AZA-H_2_O displayed markedly improved dissolution profiles. This section of the experiment investigates the dissolution process of the powder, which is conducted under identical conditions to the equilibrium solubility tests. Consequently, the pH changes and the PXRD patterns of the residues are consistent with those of the equilibrium solubility tests, and further elaboration is unnecessary. Consequently, it was concluded that these two crystalline salts enhance the dissolution rate of SPX, thereby potentially boosting its antimicrobial activity.

### 3.6. Diffusion Studies

**Figure 8:**
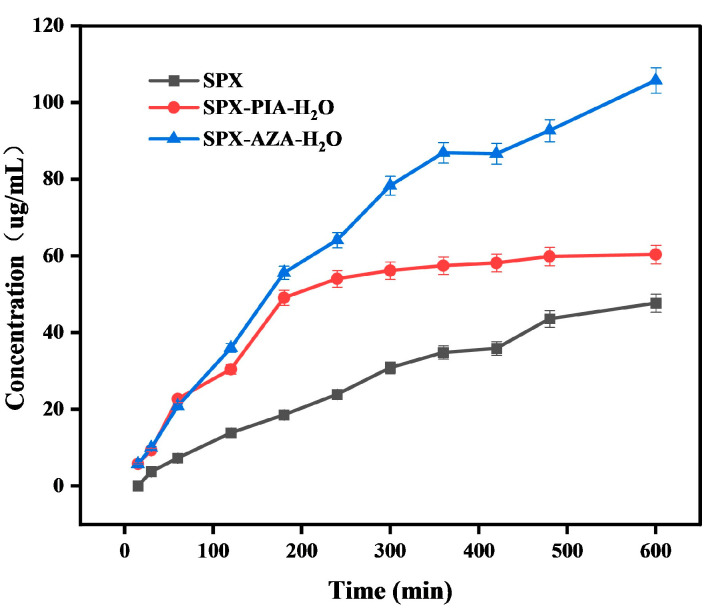
Diffusion curves of SPX and crystalline salts.

The diffusion curves and diffusion amounts of SPX, SPX-PIA-H_2_O, and SPX-AZA-H_2_O within a time period of 600 min are shown in Figure 8 and Table 1 The diffusion amounts of SPX-PIA-H_2_O (60.37 μg/mL) and SPX-AZA-H_2_O (105.76 μg/mL) are both significantly higher than that of SPX (47.67 μg/mL), indicating that the two crystalline salts can improve the solubility of SPX while enhancing diffusive ability. The pH values of the solution before and after the diffusion test are presented in Table S6. There is essentially no change in pH before and after the test, so the impact of pH on the diffusion experiment is not considered.

### 3.7. In Vitro Antimicrobial Activity

The minimum inhibitory concentration (MIC) (Table 2) indicates that AZA has a much higher antibacterial activity than PIA. Correspondingly, the antibacterial activity of the crystalline salt SPX-AZA-H_2_O is also higher than that of SPX-PIA-H_2_O. SPX-AZA-H_2_O demonstrates significantly higher inhibitory effects on *P. aeruginosa* and *B. subtilis* compared with SPX. This suggests that subsequent research could choose a coformer with better antibacterial activity (such as AZA) to synthesize crystalline salts, thereby enhancing the antimicrobial activity of the active pharmaceutical ingredients.

## 4. Discussion

In this research endeavor, we adeptly synthesized two novel crystalline salts, SPX-PIA-H_2_O and SPX-AZA-H_2_O, utilizing the solvent evaporation technique and subsequently elucidated their intricate crystal structures and crystallographic traits through the precision of single-crystal X-ray diffraction. A comprehensive battery of solid-state characterizations not only attested to the exceptional purity of these crystalline salts but also unveiled their unique thermodynamic properties, which stand in contrast to the parent compound SPX. Our property studies compellingly demonstrated that both SPX-PIA-H_2_O and SPX-AZA-H_2_O markedly augment the solubility, dissolution velocity, and permeation of SPX. The in vitro antimicrobial activity assays further disclosed that SPX-AZA-H_2_O wields a notably more potent antibacterial action against *P. aeruginosa* and *b. subtilis*, outperforming SPX, and thus constituting a monumental leap forward in our research. While this study has revealed significant findings, it is not without limitations. The mechanisms underlying the formation of the two crystalline salts were not fully and deeply elucidated, and due to the limited sample size, we could not ascertain the universal patterns of the properties of Sparfloxacin-like crystalline salts from the data obtained in this study. Despite the preliminary and limited nature of the results, this research paves the way for future in-depth exploration of the crystalline salts synthesized in this work, with the ultimate goal of expanding the clinical application range of SPX and enhancing antibacterial efficacy. This initiative is expected to provide a robust and feasible framework for the examination of drugs that are traditionally considered to be poorly soluble.

## Figures and Tables

**Table 1:** Permeation of SPX and crystalline salts.

Compound Name	Diffusion Amount (μg/mL)	Increase in Diffusion Amount (%)
SPX	47.67	--
SPX-PIA-H_2_O	60.37	26.6
SPX-AZA-H_2_O	105.76	121.9

**Table 2:** MIC values of SPX, PIA, AZA, and crystalline salts (unit: μg/mL).

Name	*E. coli*	*P. aeruginosa*	*b. subtilis*	*S. aureus*
SPX	<0.01	1	0.25	<0.02
PIA	1024	512	512	1024
AZA	32	256	512	32
SPX-PIA-H_2_O	0.06	1	0.25	0.06
SPX-AZA-H_2_O	0.03	0.25	0.06	0.016

## Data Availability

Data are contained within this article and the Supplementary Materials.

## Supplementary Materials

The following supporting information can be downloaded at: https://www.mdpi.com/article/10.3390/pharmaceutics16121519/s1, Figure S1: Molecular surface electrostatic potential energy (a) SPX; (b) PIA; (c) AZA; Figure S2: PXRD patterns of residuals after experiments under various conditions (a) SPX-PIA-H_2_O; (b) SPX-AZA-H_2_O; Table S1: Crystallographic data of SPX-PIA-H_2_O, SPX-AZA-H_2_O; Table S2: Hydrogen bond lengths (Å) and bond angles (°) of SPX-PIA-H_2_O; Table S3: Hydrogen bond lengths (Å) and bond angles (°) of SPX-AZA-H_2_O; Table S4: Equilibrium solubility values of SPX and crystalline salts; Table S5: pH value of solutions before and after the equilibrium solubility experiment; Table S6 pH value of solutions before and after the diffusion experiment. References [39,40,41,42,43] are cited in the supplementary materials.
