# Supplementary material for: Synthesis and Characterization of Two Sparfloxacin Crystalline Salts: Enhancing Solubility and In Vitro Antibacterial Activity of Sparfloxacin
Source: Pharmaceutics. 2024 Nov 26;16(12):1519. doi: 10.3390/pharmaceutics16121519 (PMC11728777; doi:10.3390/pharmaceutics16121519)
Supplement: Supplementary file 1 [file pharmaceutics-16-01519-s001.zip › pharmaceutics-3269848-supplementary.pdf]

Synthesis and characterization of two Sparfloxacin crystalline salts: Enhancing solubility and in vitro antibacterial activity of Sparfloxacin

Supplementary materials

Figure S1 Molecular surface electrostatic potential energy (a). SPX; (b). PIA; (c). AZA;

Figure S2 PXRD patterns of residuals after experiments under various conditions (a). SPX-PIA-H_2O; (b). SPX-AZA-H_2O;

Table S1 Crystallographic data of SPX-PIA-H_2O, SPX-AZA-H_2O;

Table S2 Hydrogen bond lengths (Å) and bond angles (°) of SPX-PIA-H_2O;

Table S3 Hydrogen bond lengths (Å) and bond angles (°) of SPX-AZA-H_2O;

Table S4 Equilibrium solubility values of SPX and crystalline salts;

Table S5 pH value of solutions before and after the equilibrium solubility experiment;

Table S6 pH value of solutions before and after the diffusion experiment

Molecular Electrostatic Potential (MEP)

The Gaussian 09[1] software package was utilized to optimize the structures of SPX and the two organic dicarboxylic acids using the B3LYP/6-31G*(d) basis set. The molecular electrostatic potential was visualized using the programs Multiwfn 3.8[2-4]and VMD 1.9.3[5].

The surface electrostatic potentials of SPX, PIA, and AZA were calculated separately (Figure 1). The magnitude of the electrostatic potential in different regions is displayed through different colors, with the color changing from red to white to blue as the potential decreases from high to low, and the maximum and minimum values are respectively represented by orange and blue dots. By analyzing the surface electrostatic potential and the values of the extreme points more intuitively, the possible interactions between molecules can be assessed. Combined with the analysis of the SC-XRD results, SPX has hydrogen bond acceptors and donors. The hydrogen bond acceptor is the -NH group (-24.87 kcal/mol) and the -C=O on the carboxyl group (-50.62 kcal/mol), while the hydrogen bond donor is the -NH2 group (+35.58 kcal/mol). Due to spatial reasons, the -NH2 forms intramolecular hydrogen bonds with unassociated -C=O, so only the -NH can act as a hydrogen bond acceptor, forming hydrogen bonds with the hydrogen bond donors of PIA, which are the -OH groups (49.16 kcal/mol and 49.14 kcal/mol), and AZA, which are the -OH groups (48.53 kcal/mol and 48.52 kcal/mol), thereby forming crystalline salts.

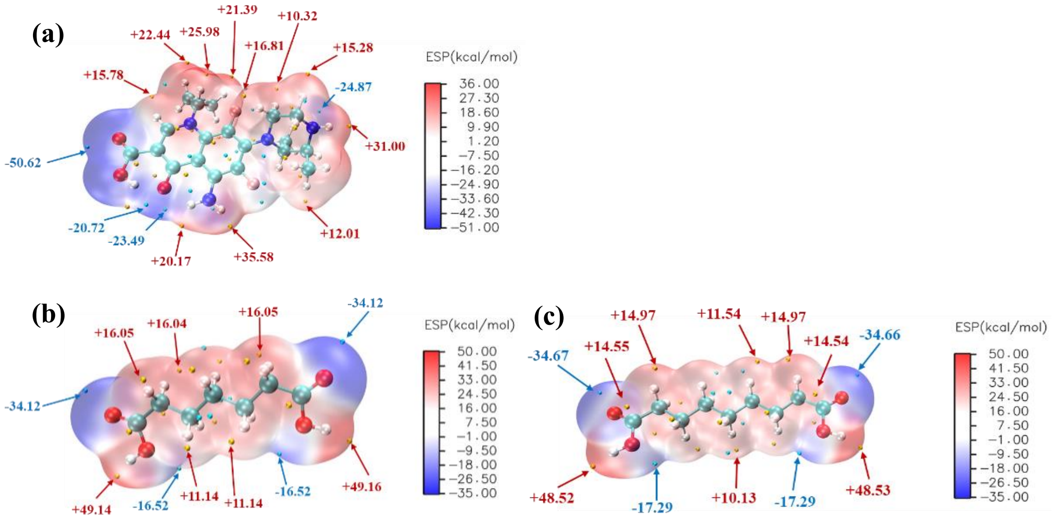

Figure S1. Molecular surface electrostatic potential energy (a). SPX; (b). PIA; (c). AZA.

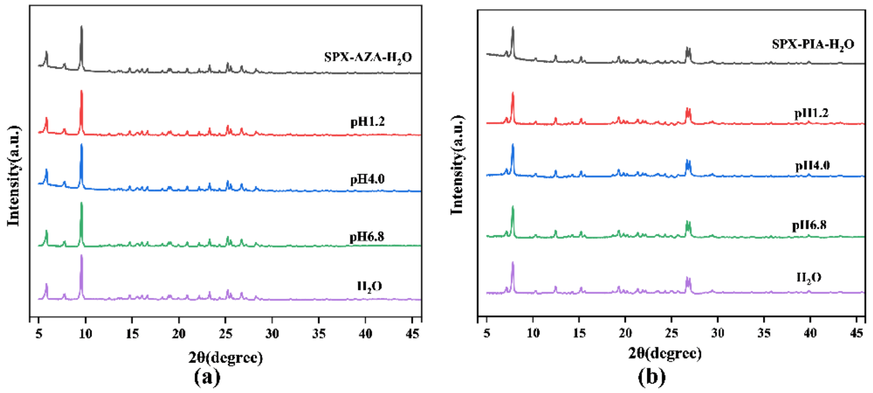

Figure S2 PXRD patterns of residuals after experiments under various conditions

Table S1 Crystallographic data of SPX-PIA-H_2O, SPX-AZA-H_2O

Compound	SPA-PIA-H_2O	SPA-AZA-H_2O
Empirical formula	$C_{45}H_{60}F_4N_8O_{12}$	$C_{47}H_{65}F_4N_8O_{12.5}$
Formula weight	981.01	1018.07
Crystal system	monoclinic	triclinic
Space group	P21/c	$P\bar{1}$
a/Å	25.2776(15)	6.9569(6)
b/Å	12.7439(7)	18.9665(18)
c/Å	14.2969(7)	19.0028(18)
α/°	90	73.338(4)
β/°	96.869(2)	87.129(4)
γ/°	90	85.843(4)
Volume/Å3	4572.5(4)	2394.7(4)
Z	4	2
ϱcalcg/cm3	1.425	1.412
Reflections collected	225354	102995
Independent reflections	10520 [Rint= 0.0403, Rsigma= 0.0131]	10989 [Rint= 0.0426, Rsigma= 0.0209]
Data/restraints/parameters	10520/0/644	10989/0/664
Goodness-of-fit on F2	1.057	1.056
Final R indexes [I>=2σ (I)]	R1 = 0.0392, wR2 = 0.1045	R1 = 0.0500, wR2 = 0.1273
Final R indexes [all data]	R1 = 0.0519, wR2 = 0.1163	R1 = 0.0640, wR2 = 0.1402
Largest diff. peak/hole / e Å-3	0.47/-0.31	0.53/-0.53

Table S2 Hydrogen bond lengths (Å) and bond angles (°) of SPX-PIA-H_2O

D-H	d(D-H)	d(H···A)	d(D···A)	<DHA	A	symmetry code
O1-H1	0.84	1.7672	2.5406	152.18	O3	
O5-H5	0.84	1.7608	2.5349	152.3	O4	
O7-H7c	0.87	1.921	2.7819	170.1	O6	+X,3/2-Y,-1/2+Z
O7-H7d	0.86	1.788	2.6440	167.5	O12	1-X,1-Y,-Z
O8-H8b	0.86	1.944	2.7993	167.5	O2	-X,1-Y,-Z
O8-H8a	0.87	1.837	2.7066	177.3	O9	+X,1/2-Y,1/2+Z
N1-H1e	0.92	1.7213	2.6371	173.25	O10	-X,1-Y,-Z
N1-H1d	0.92	1.9092	2.8206	170.48	O8	
N5-H5a	0.92	1.8607	2.7701	169.37	O11	1-X,1-Y,-Z
N5-H5b	0.92	1.8675	2.7750	168.49	O7	1-X,-1/2+Y,1/2-Z
N7-H7a	0.88	1.8875	2.6430	142.54	O3	
N7-H7b	0.88	2.029	2.8578	155.5	O11	+X,3/2-Y,1/2+Z
N3-H3b	0.89	1.96	2.6514	134.0	O4	
N3-H3a	0.89	2.0256	2.8522	153.39	O10	+X,3/2-Y,1/2+Z

Table S3 Hydrogen bond lengths (Å) and bond angles (°) of SPX-AZA-H_2O

D-H	d(D-H)	d(H···A)	d(D···A)	<DHA	A	symmetry code
O2- H2	0.84	1.74	2.5174	153.1	O3	
O10- H10	0.84	1.74	2.5172	153.4	O9	
N4- H4A	0.91	1.99	2.8009	147.3	O6	2-X,2-Y,-Z
N4- H4B	0.91	1.81	2.7141	175.0	O6	
N4-H4B	0.91	2.59	3.169	122.5	O5	
N5- H5A	0.91	1.80	2.700	169.7	O7	-X,1-Y,1-Z
N5- H5B	0.91	1.81	2.661	154.6	O8	
O4- H4C	0.87	1.95	2.796	163.8	O5	
O4-H4D	0.87	2.05	2.885	161.7	O1	2-X,2-Y,-1-Z
N2-H2A	0.88	2.14	3.010	168.0	O13	1-X,1-Y,1-Z
N2-H2B	0.88	2.01	2.664	130.3	O3	
N7-H7C	0.88	2.29	2.999	137.5	O4	-1+X,+Y,1+Z
O13-H13A	0.87	2.05	2.906	168.2	O4	
O13-H13B	0.87	1.89	2.721	157.9	O12	
C7-H7A	0.99	2.20	3.078	147.5	O12	1+X,1+Y,-2+Z
O12-H12A	0.87	2.23	3.092	173.1	O8	-X,1-Y,2-Z
O12-H12B	0.87	2.11	2.853	142.9	O11	
N7-H7D	0.88	1.96	2.643	133.0	O9	

Table S4 Equilibrium solubility values of SPX and crystalline salts

Medium	SPX	SPX-PIA-H_2O		SPX-AZA-H_2O	
	value	value	Fold Increase	value	Fold Increase
pure H_2O	0.12	0.26	2.17(+)	0.16	1.33(+)
pH1.2	0.82	1.01	1.23(+)	0.72	0.88(-)
pH4.0	0.12	0.14	1.17(+)	0.19	1.58(+)
pH6.8	0.2	0.29	1.45(+)	0.13	0.65(-)

Note: "+" signifies improved solubility, while "-" denotes reduced solubility.

Table S5 pH value of solutions before and after the equilibrium solubility experiment

before	after	
	SPX-PIA-H_2O	SPX-AZA-H_2O
1.24	1.19	1.20
4.00	3.96	3.98
6.78	6.77	6.77
H_2O（7.22）	7.86	7.58

Table S6 pH value of solutions before and after the diffusion experiment

before	after	
	SPX-PIA-H_2O	SPX-AZA-H_2O
7.42	7.42	7.48

References

[1] M.J. Frisch, G.W. Trucks, H.B. Schlegel, G.E. Scuseria, M.A. Robb, J.R. Cheeseman, G. Scalmani, V. Barone, G.A. Petersson, H. Nakatsuji, X. Li, M. Caricato, A.V. Marenich, J. Bloino, B.G. Janesko, R. Gomperts, B. Mennucci, H.P. Hratchian, J.V. Ortiz, A.F. Izmaylov, J.L. Sonnenberg, Williams, F. Ding, F. Lipparini, F. Egidi, J. Goings, B. Peng, A. Petrone, T. Henderson, D. Ranasinghe, V.G. Zakrzewski, J. Gao, N. Rega, G. Zheng, W. Liang, M. Hada, M. Ehara, K. Toyota, R. Fukuda, J. Hasegawa, M. Ishida, T. Nakajima, Y. Honda, O. Kitao, H. Nakai, T. Vreven, K. Throssell, J.A. Montgomery Jr., J.E. Peralta, F. Ogliaro, M.J. Bearpark, J.J. Heyd, E.N. Brothers, K.N. Kudin, V.N. Staroverov, T.A. Keith, R. Kobayashi, J. Normand, K. Raghavachari, A.P. Rendell, J.C. Burant, S.S. Iyengar, J. Tomasi, M. Cossi, J.M. Millam, M. Klene, C. Adamo, R. Cammi, J.W. Ochterski, R.L. Martin, K. Morokuma, O. Farkas, J.B. Foresman, D.J. Fox, Gaussian 16 Rev. A.03, Wallingford, CT, 2016.

[2] T. Lu, F. Chen, Multiwfn: A multifunctional wavefunction analyzer, Journal of computational chemistry 33(5) (2012) 580-592.

[3] J. Zhang, T. Lu, Efficient evaluation of electrostatic potential with computerized optimized code, Physical chemistry chemical physics : PCCP 23(36) (2021) 2323-2328.

[4] T. Lu, F. Chen, Quantitative analysis of molecular surface based on improved Marching Tetrahedra algorithm, Journal of molecular graphics & modelling 38 (2012) 314-323.

[5] W. Humphrey, A. Dalke, K. Schulten, VMD: Visual molecular dynamics, Journal of molecular graphics 14(1) (1996) 33-38.
